# Psychopathology and problematic social media use among children and adolescents: what possible links?

**DOI:** 10.1192/j.eurpsy.2022.702

**Published:** 2022-09-01

**Authors:** A. Ben Othman, M. Hamza, B. Amemou, S. Bourgou, A. Ben Hamouda, F. Charfi, A. Belhadj

**Affiliations:** 1 Mongi Slim University Hospital, Child And Adolescent Psychiatry Department, La Marsa, Tunisia; 2 Fattouma Bourguiba University Hospital, Psychiatry Department, Monastir, Tunisia; 3 hospital of mongi slim, Pedopsychiatry, Tunis, Tunisia

**Keywords:** Bergen Social Media Addiction Scale, Child behaviour checklist, Psychopathology, problematic use of social media

## Abstract

**Introduction:**

Social Media (SM) have recently gained substantial popularity among youth. However, the relationship between problematic use of social media (PUSM) and psychopathology in children and adolescents remains unclear.

**Objectives:**

To study in a population of children and adolescents followed in outpatient psychiatry unit, the prevalence, and psychopathological factors linked to PUSM.

**Methods:**

A descriptive study was led among child and adolescent’s psychiatry patients. Parents were asked to provide answers for the Child Behaviour Checklist (CBCL). PUSM was assessed using the Bergen Social Media Addiction Scale (BSMAS). Mental disorders’ related data were extracted from patients’ medical records.

**Results:**

Our study included 76 patients with a mean age of 14.2 ± 2.6 [11,18] years and a sex-ratio of 1. The prevalence of PUSM was estimated at 9.2% in our population according to the conservative approach, rising to 48.7% according to the liberal approach. Anxiety (32.4%) and depressive disorders (24.3%) were most prevalent among patients with PUSM. BSMAS scores were significantly higher among patients with smoking habits (p=0.03). CBCL T-scores interpretation showed internalizing and externalizing disorders among 80.3% and 64.5% patients respectively. BSMAS scores were significantly higher among patients belonging to the clinical range of all the CBCL syndrome scales, except for social problems, and among patients suffering from both internalizing and externalizing disorders (p=0.005).

**Conclusions:**

PUSM was shown to be more prevalent among clinical populations compared to healthy controls. Research has indicated a potential link between PIUSM and psychopathology; however, the significance of the correlation remains unclear.

**Disclosure:**

No significant relationships.

